# Supplementary figures and images for: Wnt10b protects cardiomyocytes against doxorubicin-induced cell death via MAPK modulation
Source: PLoS One. 2023 Oct 19;18(10):e0277747. doi: 10.1371/journal.pone.0277747 (PMC10586692; doi:10.1371/journal.pone.0277747)

## The original blot in Figure 1D

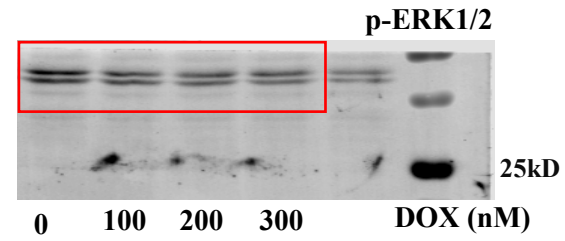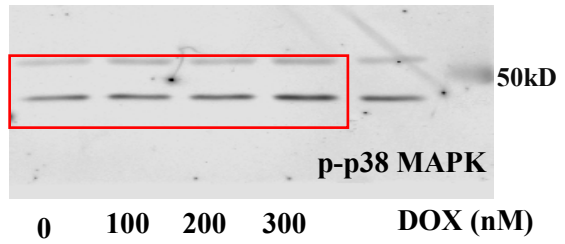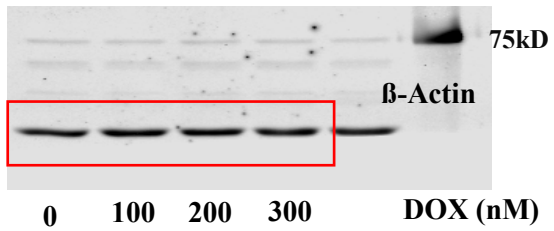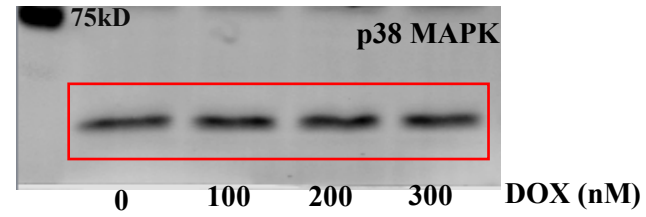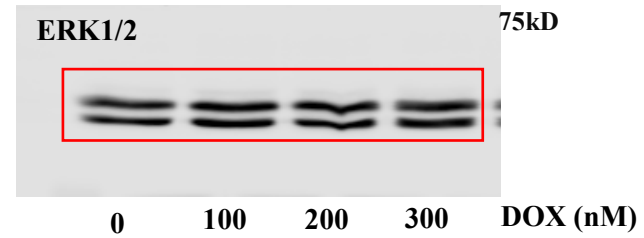

The original blot in Figure 3C

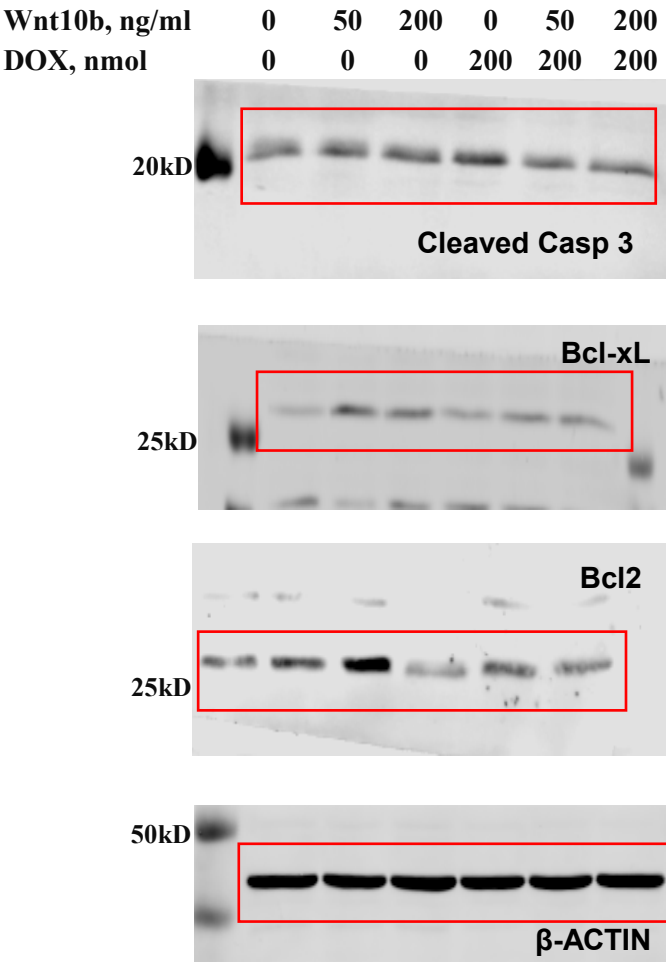

The original blot in Figure 4A

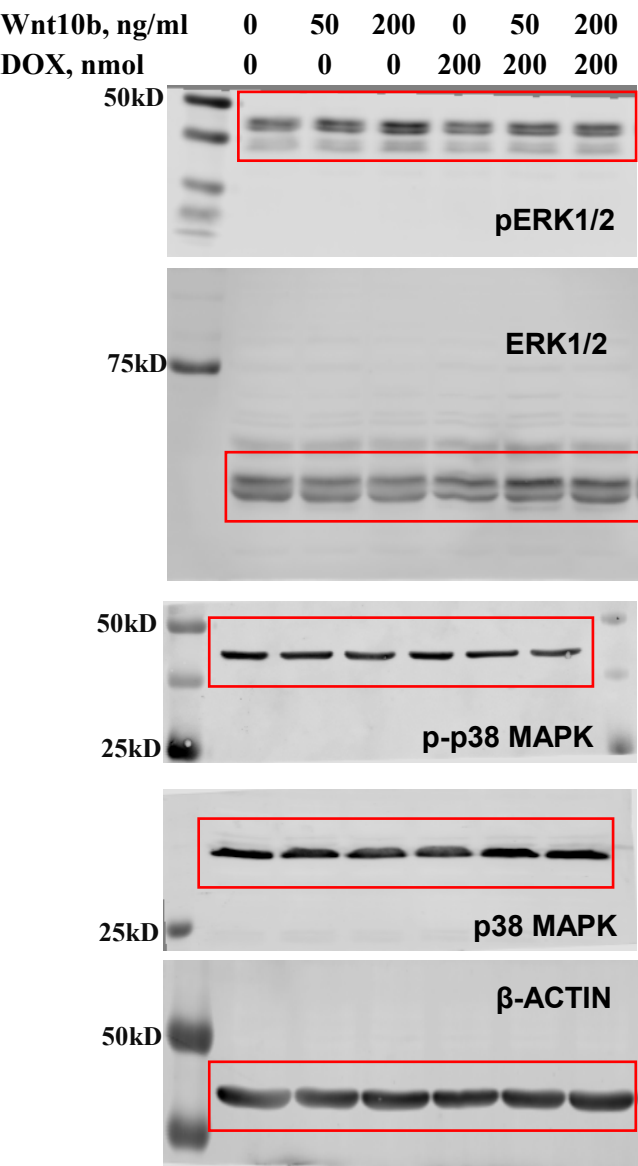

Supplement: S1 Raw images — (PDF) [file pone.0277747.s002.pdf]
